# Promoters, Key *Cis*-Regulatory Elements, and Their Potential Applications in Regulation of Cadmium (Cd) in Rice

**DOI:** 10.3390/ijms252413237

**Published:** 2024-12-10

**Authors:** Xinxin Xu, Qingxian Mo, Zebin Cai, Qing Jiang, Danman Zhou, Jicai Yi

**Affiliations:** College of Life Sciences, South China Agricultural University, Guangzhou 510642, China; 18579210455@163.com (X.X.); 13062211316@163.com (Q.M.); 15207611626@163.com (Z.C.); jiangqing547@163.com (Q.J.); zhoudm970@163.com (D.Z.)

**Keywords:** rice, cadmium (Cd), promoters, *cis*-regulatory elements (CREs)

## Abstract

Rice (*Oryza sativa*), a globally significant staple crop, is crucial for ensuring human food security due to its high yield and quality. However, the intensification of industrial activities has resulted in escalating cadmium (Cd) pollution in agricultural soils, posing a substantial threat to rice production. To address this challenge, this review comprehensively analyzes rice promoters, with a particular focus on identifying and characterizing key *cis*-regulatory elements (CREs) within them. By elucidating the roles of these CREs in regulating Cd stress response and accumulation in rice, we aim to establish a scientific foundation for developing rice varieties with reduced Cd accumulation and enhanced tolerance. Furthermore, based on the current understanding of plant promoters and their associated CREs, our study identifies several critical research directions. These include the exploration of tissue-specific and inducible promoters, as well as the discovery of novel CREs specifically involved in the mechanisms of Cd uptake, transport, and detoxification in rice. Our findings not only contribute to the existing knowledge base on genetic engineering strategies for mitigating Cd contamination in rice but pave the way for future research aimed at enhancing rice’s resilience to Cd pollution, ultimately contributing to the safeguarding of global food security.

## 1. Introduction

Cadmium (Cd) is globally recognized as a significant public health threat, exerting substantial detrimental effects on human health through the food chain. Even at low exposure levels, Cd has been linked to various adverse health outcomes, such as chronic respiratory issues, cardiovascular diseases, pulmonary ailments, and skin cancer, as reported in a Japanese study [1]. Over the past few decades, industrial activities, such as coal and waste combustion, metal smelting, fossil fuel combustion, and sewage sludge disposal, have contributed to the widespread contamination of Cd in the environment [2]. Consequently, elevated Cd concentrations have been reported in rice paddy soils worldwide, with notable variations across major rice production regions. For instance, the natural occurrence of Cd in the Earth’s crust typically ranges from 0.1 to 0.5 mg kg^−1^, whereas background levels of Cd in rice soils vary considerably across Asian countries, with reported values ranging from 0.02 to 0.6 mg kg^−1^ in India, 0.56 to 8.77 mg kg^−1^ in China, 0.136 to 0.324 mg kg^−1^ in Pakistan, and 0.83 to 4.08 mg kg^−1^ in Bangladesh [3]. Given that global rice production for the 2024–2025 period is projected to reach 527 million tons [4], and rice serves as a staple food for over half of the world’s population, meeting both nutritional and economic needs [5], the potential for daily Cd intake from rice consumption, estimated at 20 to 40 μg [6], highlights the urgency of mitigating Cd contamination in rice. The intensification of modern agricultural practices has further aggravated the Cd load in soils [7,8]. Known for its high propensity to accumulate Cd, rice represents a primary pathway of human exposure to this toxic metal. Furthermore, Cd pollution can negatively impact rice growth and development, ultimately reducing yields [9]. Therefore, a comprehensive understanding of the mechanisms governing Cd uptake and transport in rice, coupled with the development of rice varieties that exhibit low Cd accumulation and high Cd tolerance, is crucial for safeguarding food security and protecting human health.

To date, researchers have proposed various measures to reduce Cd accumulation in plants, such as chemical immobilization [10], phytoremediation [11], and water management [12,13]. While these measures can control Cd pollution in rice to a certain extent, they are difficult to apply on a large scale due to limitations, such as high cost, time-consuming efforts, and low efficiency [14]. Given the limitations of traditional control measures, researchers have begun to explore more efficient and sustainable solutions. Among them, the genetic improvement of Cd tolerance and accumulation-related genes in rice using genetic engineering has emerged as the most promising and effective approach for controlling Cd pollution in rice.

Currently, several genes related to Cd tolerance or accumulation have been identified in rice, providing abundant genetic resources for molecular breeding of Cd-tolerant and low Cd-accumulating rice varieties [9]. However, these genes have limitations, including low expression levels, weak responses to Cd stress, or a close relationship with the uptake/transport of certain essential elements, such as *OsNramp5*, *OsIRT1*, *OsIRT2*, and *OsZIP3*, which affects their application [9]. Gene expression begins with transcription, where RNA polymerase binds to the transcription start site (TSS) on the promoter and is assisted by relevant transcription factors. Furthermore, many important *cis*-regulatory elements (CREs), such as enhancers, silencers, and insulators, are mostly distributed in the promoter region [15]. These elements interact with transcription factors, playing a crucial role in conferring tissue specificity and expression strength to eukaryotic promoters [16]. They also serve as important molecular switches involved in the dynamic network regulation of gene activity, controlling many vital biological processes, including abiotic stress responses, hormone responses, and individual development [17]. Therefore, promoters can play a significant role in the field of genetic engineering related to Cd regulation in rice [18]. The development of efficient, tissue-specific inducible promoters is of great importance for cultivating new rice varieties with low Cd accumulation and high Cd tolerance, as well as for the prevention and control of Cd pollution in rice.

CREs, typically 5–15 bp in length, serve as key components in promoters, playing a central role in regulating gene expression through specific binding to transcription factors. These elements are crucial for elucidating the relationship between phenotype and genotype, as well as determining the precise temporal and spatial expression patterns of genes [19]. An in-depth exploration of the functions of CREs is of great significance in synthetic biology, crop improvement, and enhancing plant resilience under stress [20]. Especially in rice, research on *cis*-regulatory elements responsive to heavy metal (including Cd) stress has opened up new avenues for cultivating rice varieties with low Cd accumulation and high Cd tolerance.

Therefore, this paper presents a comprehensive overview of the classification, characteristics, and key *cis*-regulatory elements (CREs) of plant promoters, with a particular emphasis on rice. We delve deeply into the intricacies of tissue-specific and inducible promoters, highlighting the distinct CREs associated with each type. Furthermore, we elucidate the application of these promoters and CREs in modulating Cd tolerance and accumulation in rice, providing valuable insights into their potential utilities.

## 2. Classification and Characteristics of Promoters in Rice

As key elements regulating the spatial and temporal expression of genes, promoters play an indispensable role in plant growth and development. Based on differences in gene expression characteristics, promoters are generally classified into three types: constitutive promoters, tissue-specific promoters, and inducible promoters. Note that these classifications are not mutually exclusive, as some promoters may exhibit characteristics of multiple types.

### 2.1. Constitutive Promoters

Constitutive promoters have the ability to drive the expression of downstream genes almost uniformly and continuously in all tissues and organs of plants, maintaining a relatively stable level of mRNA and protein expression. Currently, widely used constitutive promoters in plants include the 35S promoter of the cauliflower mosaic virus (CaMV) and the promoter of the nopaline synthase gene (*Nos*) from the Ti plasmid T-DNA region of *Agrobacterium tumefaciens*. However, in monocotyledonous plants, like rice, the CaMV 35S promoter’s activity is lower, likely due to differences in gene expression regulation. Additionally, as the CaMV 35S promoter originates from a plant pathogen, its use may lead to abnormal phenotypic changes, ethical controversies, and safety issues [21]. More importantly, its activity may be inhibited under certain conditions [22]. Therefore, in rice, promoters from housekeeping genes within rice itself are preferred, such as the OsAct1 promoter [23,24] and the OsUbi1 promoter [25]. Studies have shown that the OsAct1 promoter is expressed in all tissues of rice and can efficiently drive the expression of the *HVA1* gene in rice leaves and root tissues, thereby enhancing rice tolerance to water deficit and salt stress [26]. Compared to the *ZmUbi1* gene in maize, the *OsUbi1* gene in rice is rich in pyrimidine (Py) elements in the 5′-UTR region. This structural difference makes the OsUbi1 promoter more efficient in rice, and it is widely used as a constitutive promoter in rice genetic engineering [27].

Besides the OsAct1 and OsUbi1 promoters, many other promoters that exhibit constitutive expression in rice have been identified, such as the promoters of the *APX*, *PGD1*, and *R1G1B* genes [28]. These promoters can efficiently drive the expression of downstream genes in all tissues of rice. The constitutive nature of these promoters provides a significant advantage for the widespread and abundant production of exogenous proteins across all tissues, making them indispensable tools in plant genetic engineering [29]. However, while constitutive promoters have significant advantages in expressing large amounts of foreign proteins, their non-specific drive may also lead to the production of excessive heterologous proteins or metabolites in plants, disrupting the original metabolic balance and causing a series of negative effects, such as growth retardation, early flowering, sterility, or even death [30]. To overcome this limitation, scientists have gradually shifted their focus towards the development and utilization of tissue-specific and inducible promoters [31,32,33].

### 2.2. Tissue-Specific Promoters

Tissue-specific promoters can precisely regulate the expression of downstream genes in specific organs or tissues, thereby avoiding resource waste caused by constitutive promoters. They usually exhibit regulation characteristics that match the developmental stages of plants. Based on the target plant tissues, they can be further classified into root-specific, stem-specific, leaf-specific, flower-specific, and seed-specific promoters. The potential applications of tissue-specific promoters are vast and promising. By harnessing synthetic biology techniques to integrate various CREs, researchers can achieve precise control over target gene expression in desired tissues, enabling the rational design of synthetic promoters with tissue specificity and providing powerful new tools for modulating metabolic pathways and stress responses in plants. For those seeking a comprehensive understanding of the molecular mechanisms underlying tissue-specific gene expression in plants, as well as the practical application of these mechanisms using synthetic biology approaches, a detailed review of the relevant literature is recommended [34].

#### 2.2.1. Root-Specific Promoters

As important nutritive organs that are the first to contact the soil environment in rice, roots play a central role in absorbing water, nutrients, and transporting them to other parts of the plant. Many genes related to the absorption and transport of metal elements (including heavy metals) are specifically expressed in roots. For example, the iron-regulated transporter 1 gene *OsIRT1* is induced by Fe and mainly expressed in roots [35]. The heavy metal transporter-related P1B-ATPase 3 gene *OsHMA3* primarily functions in root cells, regulating low Cd accumulation in rice [36]. The natural resistance-associated macrophage protein 5 gene *OsNramp5* is expressed mainly in roots at all growth stages of rice, with higher expression in the basal part of the roots than in the root tips [37]. Additionally, there are genes related to the transport of non-metal elements, such as the transporters low silicon rice 1 (Lsi1) and low silicon rice 2 (Lsi2), which are mainly expressed constitutively in roots and specifically transport silicon (Si) [38,39]. The phosphate transporter genes *PHOSPHATE TRANSPORTER2* (*OsPT2*) and *PHOSPHATE TRANSPORTER6* (*OsPT6*), which are related to the absorption and transport of phosphorus (P), are also specifically expressed in roots [40].

Studies have also shown that some transcription factors and enzymes involved in plant growth and development, such as the genes *QHB* and *WOX11* encoding WUSCHEL-type homeobox, are concentrated in the meristematic region of roots [41,42]. The gene *OsCSLD* encoding cellulose synthase-like D1 and the genes *OsEXPB5* and *OsEXPA17* encoding expansin are only expressed in rice root hair cells [43,44,45]. The genes *OsMADS25* and *OsMADS27* encoding rice MADS-box transcription factors have also been found to be specifically expressed in roots [46]. The gene *OsAER1*, encoding 2-alkenal reductase (*Oryza sativa* L. alkenal reductase), is mainly expressed in primary roots, lateral roots, and root hairs of rice [47]. Furthermore, the gene *OsETHE1* encoding ethylmalonic encephalopathy protein 1 (ETHE1)-like protein has the highest expression of the *GUS* gene driven by its promoter in rice roots [48].

To date, at least 17 root-specific expression genes have been identified in rice (Table 1). The promoters of these genes not only provide a rich resource pool for promoter modification and development but will also play an important role in the genetic engineering regulation of the heavy metal Cd intake and transport in rice roots.

#### 2.2.2. Green Tissue-Specific Promoters

The green tissues of rice, such as leaves and stems, are the primary sites of photosynthesis in these plants. To date, multiple genes specifically expressed in the green tissues of rice have been identified, including *cZOGT1*, *cZOGT2*, *cZOGT3*, *LEAF PANICLE2* (*LP2*), *D54O*, *DX1*, and *OrGSE*. Among them, the *cZOGT1*, *cZOGT2*, and *cZOGT3* genes are related to cZ-O-glycosyltransferase and are mainly expressed in leaves [49]. The *D54O* gene encodes a 10 kD polypeptide in photosystem II and is mainly expressed in leaves, leaf sheaths, ligules, and lemmas and paleas of young panicles [50]. The *LP2* gene encodes a leucine-rich repeat receptor kinase-like protein and is strongly expressed in photosynthetic tissues, such as leaves [51]. *DX1* and *OrGSE* are also specifically expressed in green tissues, such as leaves, leaf sheaths, stems, and panicles, and the *OrGSE* gene is induced by light [52,53]. Additionally, through the T-DNA capture line technique, researchers obtained an unknown expressed gene *Os8GSX7* with a reverse promoter (GSX7R) that can drive the expression of the *GUS* gene in green tissues except the endosperm, representing a novel reverse green tissue promoter [54]. Utilizing the promoters of these green tissue-specific expression genes (Table 2) could facilitate the transport and accumulation of Cd from roots to green tissues, thereby reducing the Cd content in grains. Therefore, these promoters have significant application potential in the genetic engineering regulation of Cd content in rice grains.

#### 2.2.3. Anther and Pollen-Specific Promoters

Anther and pollen-specific promoters primarily drive the expression of downstream genes in anthers and pollen. To date, at least 22 anther and pollen-specific expression genes have been identified in rice (Table 3). For example, the *OsLSP1*, *OsLSP2*, and *OsLSP3* genes related to the late development of rice pollen are specifically expressed only in pollen [55]. Although the application of the promoters of these genes in rice Cd regulation has not yet been reported, their potential value in rice Cd regulation-related genetic engineering warrants further exploration.

#### 2.2.4. Seed-Specific Promoters

Rice seeds serve as the primary source of human nutrition, storing large amounts of starch and protein. Seed-specific promoters can drive the specific expression of downstream genes in seeds. To date, at least 20 genes specifically expressed in rice seeds have been identified (Table 4). For example, *OsNF-YB7* is mainly expressed specifically in embryos [56]. The promoters of these genes are of great significance for seed genetic engineering, as they can be used to regulate seed development and the synthesis and storage of nutrients. Additionally, they have potential application value in regulating the accumulation of the heavy metal Cd in rice grains.

#### 2.2.5. Tissue-Specific CREs

The tissue specificity of gene expression is closely related to specific CREs present in promoters. Therefore, the identification of plant tissue-specific CREs has become an important research hotspot in the field of promoter research. To date, multiple significant tissue-specific CREs have been discovered (Table 5). For example, the *DX1* gene in rice has been identified as a green tissue-specific expression gene, and two novel green tissue-specific *cis*-regulatory elements, GSE1 and GSE2, have been found in its promoter region. GSE1 acts as a positive regulator in all green tissues (including leaves, leaf sheaths, stems, and panicles), while GSE2 specifically functions as a positive regulator in leaf sheaths and stem tissues, albeit with relatively weaker regulation [52]. Another example is the rice gene *D54O*, which encodes a 10 kD polypeptide in photosystem II. From the promoter of *D54O*, researchers identified five new tissue-specific *cis*-regulatory elements: LPSE1, LPSE2, LPSRE1, LPSRE2, and PSE1. These elements exhibit complex characteristics in regulating the tissue-specific expression of genes: LPSE1 activates the expression of the target gene in leaves and young panicles; LPSRE2 inhibits the expression of the target gene in leaves, roots, young panicles, and stems; and PSE1 specifically inhibits the expression of the target gene in young panicles and stems. Notably, LPSRE1 and LPSE2 play dual roles in tissue-specific regulation: both activate in leaves but LPSRE1 switches to inhibition in stems, while LPSE2 inhibits in young panicles and roots [50]. Subsequent investigations have identified potential protein regulatory factors that interact with these CREs. Specifically, *Os10g31330* binds exclusively to LPSE1, *Os01g10400* interacts with LPSRE1, *Os05g51180* and *Os05g37930* both associate with LPSRE2, and *Os01g01689* binds specifically to PSE1. Based on these findings, the authors postulate that the orchestration of green tissue-specific expression is likely mediated by the intricate interplay between these multiple protein factors and their corresponding CREs within the promoter regions [57]. These green tissue-specific CREs not only provide essential elements for constructing synthetic promoters with green tissue specificity but offer new insights into reducing the heavy metal content in rice grains.

### 2.3. Inducible Promoters

Inducible promoters precisely regulate gene expression in response to external stimuli like hormones, chemicals, and environmental stresses. These promoters contain multiple CREs that bind to *trans*-acting factors under specific conditions, demonstrating significant potential in the field of heavy metal cadmium (Cd) regulation. By utilizing inducible promoters, the low expression level of downstream genes can be maintained under normal conditions, thereby reducing adverse effects on plants; whereas, under specific stimuli, they can efficiently drive the expression of downstream genes, enabling precise regulation of plant growth, development, or physiological responses. Compared to constitutive or tissue-specific promoters, inducible promoters allow plants to adjust the transcription levels of specific genes in response to various signaling molecules (such as biological, physical, or chemical signals). This not only helps to reasonably reduce energy consumption in plants but avoids the negative effects of excessive gene expression products on plant growth and development. Given the importance of inducible promoters in rice genetic engineering, isolating and identifying rice inducible promoters is particularly crucial. The following sections detail the research progress on inducible promoters induced by physical factors, chemical factors, and multiple factors.

#### 2.3.1. Inducible Promoters by Physical Factors

Inducible promoters by physical factors primarily include drought-inducible and temperature-inducible promoters. Currently, eight genes induced only by drought have been identified in rice. Among them, *OsSRO1c* has been identified as a direct target gene of the transcription factor stress-responsive NAC 1 (SNAC1). SNAC1 can bind to the promoter of *OsSRO1c* and activate its expression, thereby enhancing rice tolerance to drought stress. By constructing transgenic plants expressing green fluorescent protein (GFP) driven by the promoters of *SNAC1* and *OsSRO1c* genes (*pSNAC1::GFP*, *pOsSRO1c::GFP*), it was found that *GFP* gene expression in leaves was strongly induced by drought stress. Additionally, transgenic rice plants overexpressing *SNAC1* exhibited stronger tolerance to both drought and salt stresses [59,60]. Under drought treatment, the promoters of *responsive to ABA protein 21* (*Rab21*), *water stress inducible protein 18* (*Wsi18*), *late embryogenesis abundant protein 3* (*Lea3*), *UDP-glucose 4-epimerase* (*Uge1*), *dehydration inducible protein 1* (*Dip1*), and *early drought induced protein* (*R1G1B*) genes could drive high-level expression of *GFP* in grains, with GFP fluorescence signals also significantly enhanced in green leaves, roots, and flowers, indicating that these promoters are induced by drought stress [61].

Temperature-inducible promoters can be further classified into cold-inducible and heat-inducible promoters. Since rice grows in subtropical and temperate climates, its seedling and reproductive growth stages are often threatened by cold stress [62,63]. MYBS3 is an MYB transcription factor that binds to single DNA repeats, and the *GFP* gene driven by its promoter is significantly induced by low temperature in transgenic seedlings [64]. To identify new cold-inducible promoters, researchers screened five cold-related genes (*OsABA8ox1*, *OsMYB1R35*, *OsERF104*, *OsCYP19-4*, and *OsABCB5*) from rice databases and identified their expression and promoter characteristics. RT-qPCR analysis revealed that under 4 °C treatment, the expression activities of all genes except *OsABCB5* were significantly higher than those under 10 °C or 15 °C treatments; histochemical staining of *GUS* reporter gene transformed plants further confirmed that the promoters of these genes are induced by cold stress [65].

Rice is sensitive to heat stress at various developmental stages [66]. Heat shock proteins (HSPs) play important roles in the plant life cycle, helping plants cope with both biotic and abiotic stresses [67]. The *Oshsp16.9A* gene encodes a cytoplasmic class I small heat shock protein in rice, which is induced under 41 °C heat treatment, and its transcripts decay slowly during heat treatment; the *GUS* gene expression activity driven by its promoter is stronger under heat stress [68]. Additionally, the expression characteristics of *rice cytosolic HSP70* (*OsctHSP70-1*), *OsHsfB2cp*, *PM19p*, *Hsp90p*, *rice aconitase gene* (*OsACO1*), *small heat shock protein genes* (*sHSPs*), and *OsHSP16.9C* have been reported, and the promoters of these genes can drive high expression of reporter genes such as *GUS* or *LUC* in rice under heat stress [69,70,71,72].

To date, at least 21 inducible promoters by physical factors have been identified in rice (Table 6). In the future, these promoters can be combined with genes related to Cd absorption and transport to achieve specific expression of target genes by controlling external environmental conditions (such as drought, temperature, etc.), thereby mitigating Cd stress toxicity in rice or reducing Cd accumulation in grains without affecting normal rice growth and development.

#### 2.3.2. Inducible Promoters by Chemical Factors

Inducible promoters by chemical factors can be classified into salt stress-inducible, hormone-inducible, metal ion-inducible, and non-metal ion-inducible promoters. Soil salinity is a growing global problem, and plants under salt stress face multiple challenges, such as water deficiency, ion toxicity, oxidative damage, and nutritional disorders [73]. The *OsZFP182* gene encodes a C2H2-type zinc finger protein, and under NaCl treatment, the expression of the *GUS* gene driven by its promoter was significantly enhanced in tobacco leaves and roots; when treated with MgCl_2_, CdCl_2_, ZnSO_4_, and KCl, only KCl was found to induce the expression of the *ZFP182* gene in tobacco, suggesting that the promoter of the *ZFP182* gene may specifically respond to monovalent cation stress [74]. Additionally, the expression characteristics of genes encoding the R2R3-type MYB transcription factor MULTIPASS in rice and the RAV family member OsRAV2 containing the AP2/ERF domain have been reported, showing that the expression of the *GUS* gene driven by the promoters of these genes can be significantly induced by salt stress [75,76]. Abscisic acid (ABA) plays an important role in seed development and maturation. Studies have found that the promoters of the 1Cys-peroxidase gene 1Cys-Prx and the oleosin gene *OsOle5* respond to ABA [77,78].

Particularly important, three Cd-responsive promoters have been identified in rice, including the promoters of two Tau-class glutathione S-transferase genes *OsGSTU5* and *OsGSTU37*, and the promoter of the HSP20/alpha crystallin protein family member *OsHSP18.6* gene. Under Cd stress, these promoters can drive high expression of the *GUS* gene in rice roots, stems, and leaves [79]. Arsenic (As) is a non-essential metalloid toxic to plants, often inhibiting the growth of crops such as rice and reducing yields [80]. Studies have found that the *OsARM1* gene encoding the R2R3 MYB transcription factor is induced by As stress, and under arsenite stress conditions, the expression of the *GUS* gene driven by its promoter is significantly induced in rice roots, stems, and leaves [81].

To date, at least nine genes induced by a single chemical factor have been identified in rice (Table 7). In the future, it will be necessary to further identify the elements specifically responsive to chemical factors in the promoters of these genes, providing element resources for modifying the promoters of genes related to Cd absorption or transport. Additionally, by applying exogenous chemicals, the expression of target genes can be specifically induced, thereby precisely regulating Cd accumulation in rice.

#### 2.3.3. Multi-Factor Inducible Promoters

As a crop with a short growth cycle and environmental sensitivity, rice has strict requirements for growth conditions, such as water, temperature, and light. Environmental factors, such as drought, high salinity, and extreme temperatures, not only affect rice growth and development, leading to abnormal plant morphology, reduced nutritional value of rice, but seriously affect its yield [82]. Therefore, discovering inducible promoters responsive to multiple stress factors is of great significance for cultivating new rice varieties with high tolerance. Six genes induced by multiple stresses, such as drought, high salinity, and ABA, have been successfully identified in rice, including *LIP9*, *OsNAC6*, *OsLEA14a*, *OsRAB16D*, *OsLEA3-1*, and *Oshox24* (Table 8). Under multiple stress treatments, the expression activities of the *GUS* gene driven by the promoters of these genes can be significantly induced, indicating their important roles in stress response [83]. Additionally, the response characteristics of genes, such as *SNAC2*, *Rab16A*, *OsMT-I-4b*, *OsHsfB2c*, *PM19*, *Hsp90*, *Oshsp17.3*, *Oshsp18.0*, *OsTZF1*, *OsASR1*, and *OsASR5*, to multiple stress factors have been reported. The promoters of these genes can drive high expression of reporter genes under conditions, such as drought, salt stress, ABA treatment, and heat stress, providing valuable genetic resources for cultivating drought-tolerant, salt-tolerant, and other multi-stress-resistant rice varieties [68,70,84,85,86,87,88,89]. By combining these multi-factor inducible promoters with genes related to heavy metal absorption, transport, and stress resistance, it is expected to create new multifunctional rice varieties that are resistant to multiple stresses and heavy metals.

#### 2.3.4. CREs in Inducible Promoters

With the identification of inducible promoters, the isolation and identification of key conditional inducible CREs have also become a focus of plant promoter research. Currently, most CREs identified in plant promoters are related to stress responses, such as drought, salt, temperature, light, and hormones, while the identification of the heavy metal response elements remains limited (Table 9). In the promoter region of the rice metallothionein gene *OsMT-I-4b*, researchers predicted six key elements responsive to Cu stress through bioinformatics analysis, known as copper response elements (CuREs), with the characteristic sequence 5′-GTAC-3′. Simultaneously, four elements responsive to other metal (such as Pb, Al, etc.) stresses were also discovered (metal response elements, MREs), with characteristic sequences including 5′-GAGAGCA-3′, 5′-TGCTCTC-3′, 5′-TGCAACC-3′, and 5′-TGCACCCC-3′. By constructing promoter deletion mutants and performing functional validation experiments, researchers confirmed the important roles of these elements in responding to different metal ion stresses [87]. Additionally, in the promoter of the bean stress-related gene *PvSR2*, conserved metal response elements (MREs) similar to those in the promoters of animal heavy metal-induced genes MT were found, with the characteristic sequence 5′-TGCAGGC-3′, as well as a unique metal response *cis*-regulatory element with the characteristic sequence 5′-ATTCAA-3′, which is not similar to any known metal response element sequence and may represent a new metal response *cis*-regulatory element [90]. Currently, the molecular mechanism of plant response to iron deficiency remains unknown. To reveal the *cis*-regulatory elements responsible for iron deficiency-induced expression in higher plants, researchers identified two *cis*-regulatory elements in the promoter of the barley *IDS2* gene: iron deficiency response element 1 (IDE1), with the characteristic sequence 5′-ATCAAGCATGCTTCTTGC-3′, and iron deficiency response element 2 (IDE2), with the characteristic sequence 5′-TTGAACGGCAAGTTTCACGCTGTCACT-3′. Under iron deficiency conditions, IDE1 and IDE2 synergistically drive the reporter gene to respond to iron deficiency-induced expression [91].

In future research, it is necessary to continue strengthening the identification of new CREs, especially those related to heavy metal (including Cd) regulation. By reasonably combining these elements, it is expected to design promoters with specific tissue expression patterns and the heavy metal response characteristics, thereby achieving more precise regulation of the heavy metal accumulation in rice.

## 3. Applications of Promoters and CREs in the Regulation of Cd Tolerance and Accumulation in Rice

Currently, gene editing technologies (such as CRISPR/Cas9) and overexpression technologies are widely utilized to finely regulate key transporter genes involved in Cd absorption or transport, such as *OsNramp5*, *OsHMA2*, *OsLCT1*, *OsZIP7*, and *OsCCX2*, aiming to improve rice Cd tolerance, yield, and quality. However, these Cd-related transporters often have pleiotropic functions, and the regulation of their expression may simultaneously affect the absorption and transport of other essential elements within plants. For example, while knockout of *OsNramp5*, *OsHMA2*, *OsZIP7*, and *OsCCX2* genes can reduce Cd content in rice grains, it may also lead to deficiencies in essential elements such as Zn, Ca, and Mn, thereby inhibiting rice growth and reducing yield [37,92,93,94].

The interaction between CREs in promoters and transcription factors is a crucial mechanism for regulating the spatiotemporal expression of genes. Different promoters contain various regulatory elements that can be reassembled with synthetic or natural CREs to construct synthetic promoters with specific functions (Table 10), thereby meeting specific gene expression needs. For example, as an important plant hormone, ABA plays a crucial role in plant growth, development, and stress response. Wu et al. selected the abscisic acid responsive element (ABRE) sequence (5′-ACGTGTC-3′) as the core sequence for ABA response in promoters and designed a synthetic promoter reporter system based on ABRE. This system consists of a tandem repeat sequence of six ABREs connected to a core promoter, providing an important tool for studying ABA-mediated transcriptional regulation and transcription factors interacting with ABRE [95]. To enhance plant drought tolerance, researchers identified 11 key *cis*-regulatory elements from the promoters of drought-responsive genes in soybeans and combined them with the 35S minimal promoter and a synthetic intron to design three synthetic promoters—SynP15, SynP16, and SynP18. Studies have shown that these synthetic promoters exhibit efficient drought-inducible activity, with SynP16 being the most efficient. This demonstrates that by assembling specific *cis*-regulatory elements, synthetic promoters with both stress response and tissue-specific expression patterns can be designed [96]. Additionally, three new synthetic promoters were designed, named Ap (containing a tandem repeat sequence of four ABREs), Dp (containing a tandem repeat sequence of two DREs), and ANDp (containing a tandem repeat sequence of two DREs and four ABREs). Using these promoters and selecting the cytoplasmic ABA receptor kinase 1 (*CARK1*) and regulatory components of ABA receptor 11 (*RCAR11*) as effector genes, two expression vectors with different combinations were constructed (*ANDp::CARK1*, *Dp::RCAR11-Ap::CARK1*). Studies have shown that under normal growth conditions, transgenic plants obtained from these expression vectors exhibited no significant abnormalities compared to wild-type plants, but under drought stress, all transgenic plants exhibited stronger tolerance [97]. With global climate warming, improving the heat tolerance of crops has become particularly important. Heat shock factors (HSFs) and heat shock response elements (HSEs) play crucial roles in the heat shock response (HSR) process of plants and vertebrates [98]. Maruyama et al. successfully developed a heat-inducible promoter consisting of an 18 bp fragment of an HSE from soybeans connected to a 63 bp fragment of the cor15A promoter, which can drive high expression of the *GUS* gene in *Arabidopsis* under heat shock conditions [99].

However, genetic engineering has primarily focused on unidirectional promoters, with limited designs for bidirectional promoters. Bidirectional promoters refer to DNA sequences located between adjacent and oriented gene pairs, with the ability to simultaneously drive the expression of two adjacent genes, offering greater potential. Studies have reported seven bidirectional promoters (BiGSSP1~BiGSSP7), which exhibited bidirectional expression activity when introduced into rice plants. Among them, four promoters (BiGSSP2, BiGSSP3, BiGSSP6, BiGSSP7) showed efficient and specific expression characteristics in green tissues, such as leaves, leaf sheaths, panicles, and stems [100]. To achieve precise regulation of multiple genes in plants, researchers designed a system utilizing transcription activator-like effector factors (dTALEs) to regulate synthetic TALE activation promoters (STAPs). The spatiotemporal expression patterns of these STAP promoters are determined by dTALEs factors. Each STAP promoter contains the binding sequence of dTALEs, a TATA box sequence, and upstream and downstream variable sequences to regulate the intensity of transcriptional activation. This dTALE-STAP system provides a powerful tool for fine-tuning the expression of multiple transgenes in specific developmental contexts [101].

Furthermore, as rice heavy metal ATPase 2 (OsHMA2) is involved in the transport of Zn and Cd from roots to stems, it is highly expressed in roots, nodes, and stems. Although no studies have yet reported which metal (including heavy metals)-related *cis*-regulatory elements are present in the promoter of the *OsHMA2* gene, researchers connected the promoter of the *OsHMA2* gene with the *OsHMA3* gene, enhancing the expression of *OsHMA3* in roots and stems, successfully reducing Cd content in brown rice by more than 10 times without affecting the content of other essential metals [18]. Therefore, utilizing tissue-specific promoters to target cadmium transporters provides an effective method for restricting the transfer of Cd from soil to rice grains.

With the successful application of constitutive promoters, synthetic tissue-specific promoters, and inducible promoters in rice, the absorption, transport, and accumulation of the heavy metal cadmium in rice can be precisely regulated through genetic engineering to modify or introduce specific promoters and response elements. Using tissue-specific promoters to drive the expression of genes related to Cd absorption or accumulation can restrict Cd to specific parts of the plant; alternatively, utilizing inducible promoters to drive the expression of related genes can regulate rice Cd tolerance or accumulation by applying exogenous substances or altering environmental conditions. Additionally, combining advanced technologies in synthetic biology, promoters that are both tissue-specific and inducibly responsive can be developed to achieve more precise gene regulation.

## 4. Conclusions and Outlook

**Figure 1:**
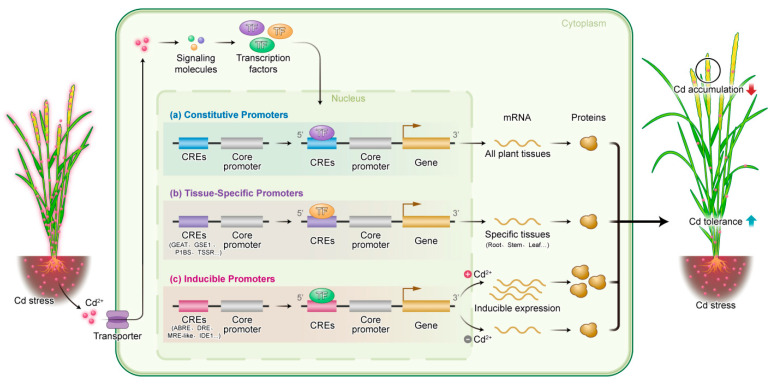
A proposed model for utilizing promoters and Cd-responsive *cis*-regulatory elements (CREs) to regulate Cd tolerance and accumulation in rice.

This review delves into the potential application prospects of rice promoters and their key CREs in the regulation of the heavy metal cadmium (Cd) (Figure 1). Plant promoters can be broadly classified into three types based on their expression characteristics: firstly, constitutive promoters, which ensure relatively stable expression levels of genes in all tissues or organs and have achieved widespread success in practical applications; secondly, tissue-specific promoters, which precisely drive gene expression in specific tissues or organs. Given the existence of specifically expressed genes in important plant organs, such as roots, stems, leaves, flowers, fruits, and seeds, in-depth identification of the promoters of these genes will lay a solid foundation for precise regulation of the heavy metal Cd in rice; finally, inducible promoters, which respond to various external factors, such as physical and chemical stimuli, or are induced by multiple factors, hold great value in genetic modification in plant genetic engineering due to their unique inducible characteristics. Additionally, both tissue-specific and inducible promoters contain special regulatory elements in their sequences. By deeply investigating the functions and regulatory mechanisms of these elements, it is expected to provide strong theoretical support for cultivating rice varieties with low Cd accumulation and high Cd tolerance.

Based on the current understanding of plant promoters and their related CREs, we have identified the following four key issues that need to be addressed in future research:

(1) Reveal the molecular basis and complete genetic regulatory mechanism of excessive Cd accumulation and transport to grains in plants.

(2) Explore how Cd interacts with other environmental stress factors (such as physical and chemical factors, including drought or other heavy metals) and the internal mechanisms by which plants maintain normal life activities under multiple stresses.

(3) Further identify undiscovered tissue-specific promoters, inducible promoters, and key CREs in plants, particularly those closely related to heavy metal responses.

(4) Design and optimize strategies to efficiently transfer Cd to non-edible parts of rice plants, thereby effectively reducing Cd content in edible parts while ensuring that plants maintain necessary nutritional homeostasis.

Figure 1 elucidates the sophisticated mechanism through which diverse promoter types and their associated Cd-responsive *cis*-regulatory elements (CREs) orchestrate Cd tolerance and accumulation in plants, using rice as a representative model. Upon exposure to Cd stress, rice plants generate intracellular signal molecules that activate specific transcription factors. These transcription factors subsequently recognize and bind to single or multiple CREs, thereby modulating the transcription of downstream target genes. This regulatory framework enables plants to fine-tune their gene expression in response to Cd stress, effectively limiting Cd accumulation in rice grains and enhancing plant resilience. (a) Constitutive promoters: These promoters ensure the constitutive expression of target genes across all plant tissues, making them ideal for a ubiquitous response to Cd stress. This global expression pattern facilitates rapid plant adaptation to Cd exposure. (b) Tissue-specific promoters: By restricting gene expression to specific tissues, such as roots, stems, or leaves, these promoters play a crucial role in sequestering Cd within these tissues. This localization strategy minimizes Cd translocation to sensitive tissues like grains, thereby preserving plant growth and developmental integrity. (c) Inducible promoters: These promoters are responsive to external stimuli or environmental cues, providing temporal and conditional control over gene transcription. This flexibility allows plants to adopt a more precise and adaptive regulatory strategy in response to Cd stress. Through the strategic combination of different promoters and CREs via genetic engineering, precise regulation of plant responses to Cd stress and tolerance can be achieved. This approach not only reduces Cd content in grains but enhances overall plant safety and growth performance, contributing to the development of Cd-resistant rice varieties with improved agricultural sustainability and food security. 

## Figures and Tables

**Table 1:** Root-specific expression genes in rice.

Gene	Gene ID (MSU_RGAP)	Gene ID (RAP)	Expression Location	Reference
*OsIRT1*	LOC_Os03g46470	Os03g0667500	Root	https://doi.org/10.1093/jxb/erf004
*OsHMA3*	LOC_Os07g12900	Os07g0232900	Root	https://doi.org/10.1073/pnas.1005396107
*Nramp5*	LOC_Os07g15370	Os07g0257200	Root	https://doi.org/10.1105/tpc.112.096925
*Lsi1*	LOC_Os02g51110	Os02g0745100	Lateral root	https://doi.org/10.1038/nature04590
*Lsi2*	LOC_Os03g01700	Os03g0107300	Lateral root	https://doi.org/10.1038/nature05964
*OsPT2*	LOC_Os03g05640	Os03g0150800	Stele of primary and lateral roots	https://doi.org/10.1111/j.1365-313X.2008.03726.x
*OsPT6*	LOC_Os08g45000	Os08g0564000	Both epidermal and cortical cells of the younger primary	https://doi.org/10.1111/j.1365-313X.2008.03726.x
*QHB*	LOC_Os01g63510	Os01g0854500	Root tip	https://doi.org/10.1046/j.1365-313x.2003.01816.x
*WOX11*	LOC_Os07g48560	Os07g0684900	Root tip and cell division zones of primary and lateral roots	https://doi.org/10.1105/tpc.108.061655
*OsCSLD1*	LOC_Os10g42750	Os10g0578200	Root hair	https://doi.org/10.1104/pp.106.091546
*OsEXPB5*	LOC_Os04g46650	Os04g0552200	Root hair	https://doi.org/10.1007/s10059-010-0127-7
*OsEXPA17*	LOC_Os06g01920	Os06g0108600	Root hair	https://doi.org/10.1111/j.1365-313X.2011.04533.x
*OsMADS25*	LOC_Os04g23910	Os04g0304400	Root’s central cylinder	https://doi.org/10.1016/j.gep.2013.02.004
*OsMADS27*	LOC_Os02g36924	Os02g0579600	Root’s central cylinder	https://doi.org/10.1016/j.gep.2013.02.004
*OsETHE1*	LOC_Os01g47690	Os01g0667200	Root	https://doi.org/10.1111/ppl.12147
*OsAER1*			Root	https://doi.org/10.1071/FP18237

(MSU_RGAP: https://rice.uga.edu/; accessed on 9 December 2023; RAP: https://rapdb.dna.affrc.go.jp/index.html; accessed on 9 December 2023).

**Table 2:** Green tissue-specific expression genes in rice.

Gene	Gene ID (MSU_RGAP)	Gene ID (RAP)	Expression Location	Reference
*cZOGT1*	LOC_Os04g46980	Os04g0556500	Leaf blade	https://doi.org/10.1104/pp.112.196733
*cZOGT2*	LOC_Os04g46990	Os04g0556600	Leaf blade	https://doi.org/10.1104/pp.112.196733
*OsCHLH*	LOC_Os03g20700	Os03g0323200	Green tissue	https://doi.org/10.1093/pcp/pcg064
*D54O*	LOC_Os08g10020	Os08g0200300	Green tissue	https://doi.org/10.1111/j.1467-7652.2007.00271.x
*LP2*	LOC_Os02g40240	Os02g0615800	Green tissue	https://doi.org/10.1111/j.1467-7652.2009.00449.x
*DX1*	LOC_Os12g33120	Os12g0515800	Green tissue	https://doi.org/10.1007/s00299-012-1238-8
*OrGSE*			Green tissue	https://doi.org/10.3390/ijms19072009
*Os8GSX7*	LOC_Os01g35580	Os01g0538000	Green tissue	https://doi.org/10.3390/biology11081092

(MSU_RGAP: https://rice.uga.edu/; accessed on 9 December 2023; RAP: https://rapdb.dna.affrc.go.jp/index.html; accessed on 9 December 2023).

**Table 3:** Anther and pollen-specific expression genes in rice.

Gene	Gene ID (MSU_RGAP)	Gene ID (RAP)	Expression Location	Reference
*OsCP1*	LOC_Os04g57490	Os04g0670500	Anther	https://doi.org/10.1023/B:PLAN.0000040904.15329.29
*OsSCP1*	LOC_Os01g06490	Os01g0158200	Anther	https://doi.org/10.1007/s00299-005-0077-2
*OsSCP2*	LOC_Os01g11670	Os01g0215100	Anther	https://doi.org/10.1007/s00299-005-0077-2
*OsSCP3*	LOC_Os01g22980	Os01g0332800	Anther	https://doi.org/10.1007/s00299-005-0077-2
*CYP703A3*	LOC_Os08g03682	Os08g0131100	Anther	https://doi.org/10.1105/tpc.108.062935
*KAR*	LOC_Os12g13930	Os12g0242700	Anther	https://doi.org/10.1105/tpc.108.062935
*CYP704B2*	LOC_Os03g07250	Os03g0168600	Anther	https://doi.org/10.1105/tpc.109.070326
*OsLSP4*	LOC_Os02g50770	Os02g0741200	Anther	https://doi.org/10.1007/s00497-014-0239-x
*OsLSP6*	LOC_Os01g69020	Os01g0919200	Anther	https://doi.org/10.1007/s00497-014-0239-x
*OsLSP7*	LOC_Os05g46530	Os05g0543000	Anther	https://doi.org/10.1007/s00497-014-0239-x
*OsLSP8*	LOC_Os07g14340	Os07g0247000	Anther	https://doi.org/10.1007/s00497-014-0239-x
*OsLSP9*	LOC_Os04g25190	Os04g0317800	Anther	https://doi.org/10.1007/s00497-014-0239-x
*OsLSP1*	LOC_Os02g09540	Os02g0188600	Anther	https://doi.org/10.1111/jipb.12912
*OsLSP2*	LOC_Os01g68540	Os01g0913600	Anther	https://doi.org/10.1111/jipb.12912
*OsLSP3*	LOC_Os10g21110	Os10g0351700	Anther	https://doi.org/10.1111/jipb.12912
*OsSUT3*	LOC_Os10g26470	Os10g0404500	Anther	https://doi.org/10.3390/ijms21061909
*OsHFP*	LOC_Os04g13540	Os04g0213100	Anther-specific	https://doi.org/10.1016/j.bbrc.2012.08.088
*OsLTP6*	LOC_Os10g05720	Os10g0148000	Anther-specific	https://doi.org/10.1038/nrg3583
*OsIPP3*	LOC_Os05g46530	Os05g0543000	Anther-specific	https://doi.org/10.1007/s00497-015-0264-4
*RTS*	LOC_Os01g70440	Os01g0929600	Anther’s tapetum	https://doi.org/10.1007/s11103-006-9031-0
*OSRIP18*	LOC_Os07g37090	Os07g0556800	Anther’s tapetum	https://doi.org/10.1007/s11103-006-9031-0
*RIP1*	LOC_Os12g03822	Os12g0132400	Pollen	https://doi.org/10.1093/pcp/pcl013
*MADS62*	LOC_Os08g38590	Os08g0494100	Pollen	https://doi.org/10.1038/nrg3583
*MADS63*	LOC_Os06g11970	Os06g0223300	Pollen	https://doi.org/10.1038/nrg3583
*MADS68*	LOC_Os11g43740	Os11g0658700	Pollen	https://doi.org/10.1038/nrg3583
*OsUgp2*	LOC_Os02g02560	Os02g0117700	Pollen-specific	https://doi.org/10.1007/s11033-010-0553-9
*OSIPA*			Pollen-specific	https://doi.org/10.1007/s12033-010-9347-5

(MSU_RGAP: https://rice.uga.edu/; accessed on 9 December 2023; RAP: https://rapdb.dna.affrc.go.jp/index.html; accessed on 9 December 2023).

**Table 4:** Seed-specific expression genes in rice.

Gene	Gene ID (MSU_RGAP)	Gene ID (RAP)	Expression Location	Reference
*RSUS3*	LOC_Os07g42490	Os07g0616800	Endosperm	https://doi.org/10.1007/s00299-006-0158-x
*GluA-1*	LOC_Os01g55690	Os01g0762500	Endosperm	https://doi.org/10.1093/jxb/ern110
*GluA-2*	LOC_Os10g26060	Os10g0400200	Endosperm	https://doi.org/10.1093/jxb/ern110
*GluA-3*	LOC_Os03g31360	Os03g0427300	Endosperm	https://doi.org/10.1093/jxb/ern110
*GluB-5*	LOC_Os02g16820	Os02g0268100	Endosperm	https://doi.org/10.1093/jxb/ern110
*GluB-3*			Endosperm	https://doi.org/10.1093/jxb/ern110
*GluC*	LOC_Os02g25640	Os02g0453600	Endosperm	https://doi.org/10.1093/jxb/ern110 https://doi.org/10.1007/s00122-010-1386-6
*OsSSII-3*	LOC_Os06g12450	Os06g0229800	Endosperm	https://doi.org/10.1002/jsfa.6230
*GluB-1*	LOC_Os02g15178/ LOC_Os02g15169	Os02g0249800/ Os02g0249900	Endosperm close to the embryo	https://doi.org/10.1111/j.1467-7652.2004.00055.x https://doi.org/10.1073/pnas.0503428102
*GluB-2*	LOC_Os02g15070	Os02g0248800	Endosperm	https://doi.org/10.1111/j.1467-7652.2004.00055.x
*GluB-4*	LOC_Os02g16830	Os02g0268300	Endosperm	https://doi.org/10.1111/j.1467-7652.2004.00055.x
*PG5a*	LOC_Os05g26386/ LOC_Os05g26377	Os05g0329100	Outer portion of the endosperm	https://doi.org/10.1111/j.1467-7652.2004.00055.x
*Glb-1*	LOC_Os05g41970	Os05g0499100	Inner starchy endosperm tissue	https://doi.org/10.1111/j.1467-7652.2004.00055.x
*AL1*	LOC_Os01g28474	Os01g0382200	Aleurone layer of endosperm	https://doi.org/10.1007/s11103-011-9765-1
*OLE18*	LOC_Os03g49190	Os03g0699000	Embryo and aleurone layer	https://doi.org/10.1111/j.1467-7652.2004.00055.x
*1Cys-Prx*	LOC_Os07g44430	Os07g0638300	Embryo	https://doi.org/10.1016/j.bbrc.2011.03.120
*OsNF-YB7*	LOC_Os02g49370	Os02g0725700	Embryo	https://doi.org/10.1111/tpj.15230
*OsRRM*	LOC_Os09g34070	Os09g0516300	Endosperm	https://doi.org/10.1038/cr.2007.43
*OsNF-YB1*	LOC_Os02g49410	Os02g0725900	Endosperm	https://doi.org/10.1016/j.gene.2014.08.059

(MSU_RGAP: https://rice.uga.edu/; accessed on 9 December 2023; RAP: https://rapdb.dna.affrc.go.jp/index.html; accessed on 9 December 2023).

**Table 5:** Tissue-specific *cis*-regulatory elements (CREs) in plant promoters [58].

Plant Tissue	*Cis*-Acting Element
Root	RSE, ROOTMOTIFTAPOX1, RHERPATEXPA7, tef-box, TGA1a, MYCCONSENSUSAT, SORLIP1AT, RAV1AAT, LEAFYATAG, SURECOREATSULTR11, P1BS, SP8BFIBSP8AIB, 141NTG13, AUXREPSIAA4, OSE 1-ROOTNODULE, OSE2ROOTNODULE, WUSATAg, and XYLAT
Stem/Tuber	ABF, as-2-box, BBOXSITE1STPAT, TSSR
Leaf	GATFLK, LPSE1, PSE1, GRA, RAV1, TCCAAAA motifs
Chlorenchyma	GEAT, GSE1, and GSE2
Other tissues (vascular bundle, guard cell, etc.)	ACIIIPVPAL2, BS1, DOF binding sites, LPSRE1
Flower	AGAMOUSAT, AGL 1, CArG-box, TACPyAT and CHS promoter core fragments (PCCHS, LCHS), GATA-box, CACT-box, CACG-box, MYBPLANT, MYB26PS
Pollen	PS region, POLLEN1LELAT52, POLLEN1, GTGANTG10, VOZ binding sequence, GTGA-box, telo-box, A9 and TA29 promoter fragments, anther box
Fruitage	TAAAG motif, E-box, SEF binding site, AGTTAGG, TGTCACA and SlHDC-A core promoter regions
Grain	Skn-1, RY motif, O2 site, E-box, AACACORE, ABAD, AMYBOX1, CAREOSREP1, EM, ESP, GLMHVCHORD, Sph element, TGACGT motif, A 27 zn, Glb 1, GCN 4 motif, CANBNNAPA, CATGTAA

**Table 6:** Physically induced expression genes in rice.

Gene	Gene ID (MSU_RGAP)	Gene ID (RAP)	Expression Location	Reference
*SNAC1*	LOC_Os03g60080	Os03g0815100	Drought-inducible	https://doi.org/10.1073/pnas.0604882103
*OsSRO1c*	LOC_Os03g12820	Os03g0230300	Drought-inducible	https://doi.org/10.1093/jxb/ers349
*Rab21*	LOC_Os11g26790	Os11g0454300	Drought-inducible	https://doi.org/10.1007/s00425-010-1212-z
*Wsi18*	LOC_Os01g50910	Os01g0705200	Drought-inducible	https://doi.org/10.1007/s00425-010-1212-z
*Lea3*	LOC_Os05g46480	Os05g0542500	Drought-inducible	https://doi.org/10.1007/s00425-010-1212-z
*Uge1*	LOC_Os05g51670	Os05g0595100	Drought-inducible	https://doi.org/10.1007/s00425-010-1212-z
*Dip1*	LOC_Os02g44870	Os02g0669100	Drought-inducible	https://doi.org/10.1007/s00425-010-1212-z
*R1G1B*	LOC_Os05g04700	Os05g0138300	Drought-inducible	https://doi.org/10.1007/s00425-010-1212-z
*MYBS3*	LOC_Os10g41200	Os10g0561400	Cold-inducible	https://doi.org/10.1104/pp.110.153015
*OsABA8ox1*	LOC_Os02g47470	Os02g0703600	Cold-inducible	https://doi.org/10.1007/s00425-017-2765-x
*OsMYB1R35*	LOC_Os04g49450	Os04g0583900	Cold-inducible	https://doi.org/10.1007/s00425-017-2765-x
*OsERF104*	LOC_Os08g36920	Os08g0474000	Cold-inducible	https://doi.org/10.1007/s00425-017-2765-x
*OsCYP19-4*	LOC_Os06g49470	Os06g0708400	Cold-inducible	https://doi.org/10.1007/s00425-017-2765-x
*OsABCB5*	LOC_Os01g50100	Os01g0695800	Cold-inducible	https://doi.org/10.1007/s00425-017-2765-x
*Oshsp16.9A*	LOC_Os01g04370	Os01g0136100	Heat-inducible	https://doi.org/10.1007/s11103-004-5182-z
*OsctHsp70-1*	LOC_Os05g38530	Os05g0460000	Heat-inducible	https://doi.org/10.1007/s10142-013-0331-6
*OsHsfB2c*	LOC_Os09g35790	Os09g0526600	Heat-inducible	https://doi.org/10.1155/2013/397401
*AWPM-19*	LOC_Os05g31670	Os05g0381400	Heat-inducible	https://doi.org/10.1155/2013/397401
*Hsp90*	LOC_Os04g01740	Os04g0107900	Heat-inducible	https://doi.org/10.1155/2013/397401
*OsACO1*	LOC_Os03g04410	Os03g0136900	Heat-inducible	https://doi.org/10.1016/j.plantsci.2015.01.003
*OsHSP16.9C*	LOC_Os01g04360	Os01g0136000	Heat-inducible	https://doi.org/10.1016/j.bbrc.2016.09.056

(MSU_RGAP: https://rice.uga.edu/; accessed on 9 December 2023; RAP: https://rapdb.dna.affrc.go.jp/index.html; accessed on 9 December 2023).

**Table 7:** Chemically induced expression genes in rice.

Gene	Gene ID (MSU_RGAP)	Gene ID (RAP)	Expression Location	Reference
*ZFP182*	LOC_Os03g60560	Os03g0820300	Salt-inducible	https://doi.org/10.1007/s11033-010-0553-9
*OsRAV2*	LOC_Os01g04800	Os01g0141000	Salt-inducible	https://doi.org/10.1007/s11103-015-0393-z
*OsMPS*	LOC_Os02g40530	Os02g0618400	Salt-inducible	https://doi.org/10.1111/tpj.12286
*1Cys-Prx*	LOC_Os07g44430	Os07g0638300	ABA-inducible	https://doi.org/10.1016/j.bbrc.2011.03.120
*OsOle5*	LOC_Os03g49190	Os03g0699000	ABA-inducible	https://doi.org/10.1016/j.jplph.2017.04.015
*OsHSP18.6*	LOC Os03g16030	Os03g0267000	Cd-inducible	https://doi.org/10.1016/j.jbiotec.2015.09.037
*OsGSTU5*	LOC Os09g20220	Os09g0367700	Cd-inducible	https://doi.org/10.1016/j.jbiotec.2015.09.037
*OsGSTU37*	LOC Os01g72150	Os01g0949900	Cd-inducible	https://doi.org/10.1016/j.jbiotec.2015.09.037
*OsARM1*	LOC_Os05g37060	Os05g0442400	As-inducible	https://doi.org/10.3389/fpls.2017.01868

(MSU_RGAP: https://rice.uga.edu/; accessed on 9 December 2023; RAP: https://rapdb.dna.affrc.go.jp/index.html; accessed on 9 December 2023).

**Table 8:** Multi-factor induced expression genes in rice.

Gene	Gene ID (MSU_RGAP)	Gene ID (RAP)	Expression Location	Reference
*LIP9*	LOC_Os02g44870	Os02g0669100	Drought, salinity, and ABA stress-inducible	https://doi.org/10.1007/s00425-013-1960-7
*OsNAC6*	LOC_Os01g66120	Os01g0884300	Drought, salinity, and ABA stress-inducible	https://doi.org/10.1007/s00425-013-1960-7
*OsLEA14a*	LOC_Os01g50910	Os01g0705200	Drought, salinity, and ABA stress-inducible	https://doi.org/10.1007/s00425-013-1960-7
*OsRAB16D*	LOC_Os11g26780	Os11g0454200	Drought, salinity, and ABA stress-inducible	https://doi.org/10.1007/s00425-013-1960-7
*Oshox24*	LOC_Os02g43330	Os02g0649300	Drought, salinity, and ABA stress-inducible	https://doi.org/10.1007/s00425-013-1960-7
*OsLEA3-1*	LOC_Os05g46480	Os05g0542500	Drought, salinity, and ABA stress-inducible	https://doi.org/10.1007/s00425-013-1960-7; https://doi.org/10.1007/s00122-007-0538-9
*SNAC2*	LOC_Os01g66120	Os01g0884300	Drought, salt, cold, wound stress-inducible	https://doi.org/10.1007/s11103-008-9309-5
*Rab16A/RAB21*	LOC_Os11g26790	Os11g0454300	Salt and ABA-inducible	https://doi.org/10.1007/s00299-011-1072-4 https://doi.org/10.1007/s11248-009-9263-2
*OsMT-I-4b*	LOC_Os12g38051	Os12g0568200	Drought, salt, ABA, heavy metal and dark stress-inducible	https://doi.org/10.1111/j.1744-7909.2010.00966.x
*OsHsfB2c*	LOC_Os09g35790	Os09g0526600	High heat stress-inducible, weak drought stress-inducible	https://doi.org/10.1155/2013/397401
*HSP90*	LOC_Os02g04650	Os02g0139100	High heat stress-inducible, weak drought stress-inducible	https://doi.org/10.1155/2013/397401
*PM19*	LOC_Os05g31670	Os05g0381400	High heat stress-inducible, weak drought stress-inducible	https://doi.org/10.1155/2013/397401
*Oshsp17.3*	LOC_Os03g16020	Os03g0266900	Heat and Aze treatment-inducible	https://doi.org/10.1007/s11103-004-5182-z
*Oshsp18.0*	LOC_Os03g16030	Os03g0267000	Heat and Aze treatment-inducible	https://doi.org/10.1007/s11103-004-5182-z
*OsTZF1*	LOC_Os05g10670	Os05g0195200	ABA, NaCl inducible	https://doi.org/10.1104/pp.112.205385
*OsASR1*	LOC_Os01g72900	Os01g0959100	Dehydration and low temperature-inducible	https://doi.org/10.1007/s00299-013-1512-4
*OsASR5*	LOC_Os01g72900	Os01g0959100	Dehydration and low temperature-inducible	https://doi.org/10.1007/s00299-013-1512-4

(MSU_RGAP: https://rice.uga.edu/; accessed on 9 December 2023; RAP: https://rapdb.dna.affrc.go.jp/index.html; accessed on 9 December 2023).

**Table 9:** CREs in plant promoters and their responsive stress types.

Response Type		*Cis*-Acting Element
Physical factor	Drought stress	RSE, ROOTMOTIFTAPOX1, RHERPATEXPA7, tef-box, TGA1a, MYCCONSENSUSAT, SORLIP1AT, RAV1AAT, LEAFYATAG, SURECOREATSULTR11, P1BS (Root hairs), SP8BFIBSP8AIB (Block root), 141NTG13 (Root tip), AUXREPSIAA4 (Root tip), OSE 1-ROOTNODULE (Root nodule-infected cells), OSE2ROOTNODULE (Root nodule-infected cells), WUSATAg (Apical meristem), and XYLAT (Core xylem)
	Temperature stress	DRE, CARGATCONSENSUS, CBF, CRT, LTRE, TCA-like, CAT-box, HSE, and GAATTC
	Drought stress	ABAD, ABRE, ACGT, DRE, EMBP 1, MYB 1, MYB 2 and SRENTTTO1, NACR/HDZFR, MYBR/MYR, MBS, Erd 1, TC-rich repeat, GT1 motif, E-box, and STRE
Chemical factor	Salt stress	GT-1
	Auxin	RE, AuxRE, TGA elements, conjugation elements CE1, and CE3
	Abscisic acid	ABRE, ABRC, ABRERATCAL, ABRELATERD1, coupled elements CEs, and STRE
	Phytokinin	CANBNNAPA, MYBGAHV, and CARG 1
	Gibberellin	GARE motif and as-1-like
	Jasmonic	20NTNTNOS, JASE 1, JERE, T/G-box, ACG motif, and as-1-like
	Ethylene	EIN 3, EREGCC, ERE, GCCCORE, YREGIONNTPRB1B, and Y regions
	Salicylic acid	AS1, LS5, LS7, SARECAMV, and TCA 1 motifs
	Heavy metal induction	MRE-like
	Iron deficiency	IDE1, IDE2
	Cu	GTAC
Multiple factors		LTRE, CRT, MYBR, DRE, MYC-like, G-box, MYCR, as-1-like, TC-rich repeats, W-box, PRE2, H-box, E-box,

**Table 10:** Some synthetic promoters designed in plants.

Synthetic Promoter Name	*cis*-Element	Expression Pattern	Function	Reference
*6×ABRE SP*	ABRE	ABAt inducible	ABA inducible	https://doi.org/10.1104/pp.18.00401
*Ap*; *Dp*; *ANDp*	DRE; ABRE; TATA; CAAT	Stress inducible	ABA/drought inducible	https://doi.org/10.3390/ijms19071945
*ZmDRO1B^73^*	cis elements from maize	Mainly in roots	ABA/drought inducible	https://doi.org/10.1111/pbi.13889
	TGAC; ACGT		Salt, ABA inducible	https://doi.org/10.1038/s41598-019-38757-7
*SP-DD*; *SP-FF*; *SP-FFDD*	F element; D box		Ascochyta rabiei, JA/SA inducible	https://doi.org/10.1186/s13568-019-0919-x
*BL1*; *BL2*	AGATA; AGATG, etc.	Stress inducible	Drought stress inducible	https://doi.org/10.3839/jabc.2021.007
*SP1*; *SP2*; *SP3*	MYBPLANT; MYBPZM; E2FANTRNR; RAV1AT; SORLIP5		Drought inducible	https://doi.org/10.1071/FP21314
*SynP15*; *SynP16*; *SynP18*;	ABRE; MYB; E2F-VARIANT, etc.	Root specific	Root specific, drought inducible	https://doi.org/10.3390/ijms21041357
*pKANADI- Motif^SYNTHETIC^*; *pKANADI- Motif^SYNTHETIC^*; *pWRKY- Motif^SYNTHETIC^*;		Root specific	Cell type-specific (Root epidermis and cortical cell-specific)	https://doi.org/10.1105/tpc.20.00154
*STAP*	EBE; TATA, etc.	Controlled and specific expression	Controllable-specific cell-type expression	https://doi.org/10.1111/pbi.13864
*cor15A+HSE*	HSE	Specific in individual cells of plant seedlings or roots	Cell type-specific	https://doi.org/10.1111/tpj.13420
*GSSP1*; *GSSP3*; *GSSP5*; *GSSP6*; *GSSP7*	G Box; GT1; GEAT	Green tissue specific	Green tissue specific	https://doi.org/10.1038/srep18256
*BiGSSP2*; *BiGSSP3*; *BiGSSP6*; *BiGSSP7*;	Rca; Ppask; LP2; G box; GATA; GEAT, etc.	Green tissue specific	Bidirectional expression in green tissues	https://doi.org/10.1111/pbi.13231
